# Exploring the genetic and molecular basis of differences in multiple myeloma of individuals of African and European descent

**DOI:** 10.1038/s41418-023-01236-8

**Published:** 2023-11-24

**Authors:** Arnold J. Levine, John D. Carpten, Maureen Murphy, Pierre Hainaut

**Affiliations:** 1 Simons Center for Systems Biology, Institute for Advanced Study, Princeton, NJ USA; 2 City of Hope Comprehensive Cancer Center, Duarte, CA USA; 3 The Wistar Institute, Philadelphia, PA USA; 4 Institute for Advanced Biosciences, Université Grenoble Alpes, Grenoble, France

**Keywords:** Cancer genetics, Haematological diseases

## Abstract

Multiple Myeloma is a typical example of a neoplasm that shows significant differences in incidence, age of onset, type, and frequency of genetic alterations between patients of African and European ancestry. This perspective explores the hypothesis that both genetic polymorphisms and spontaneous somatic mutations in the *TP53* tumor suppressor gene are determinants of these differences. In the US, the rates of occurrence of MM are at least twice as high in African Americans (AA) as in Caucasian Americans (CA). Strikingly, somatic *TP53* mutations occur in large excess (at least 4–6-fold) in CA versus AA. On the other hand, *TP53* contains polymorphisms specifying amino-acid differences that are under natural selection by the latitude of a population and have evolved during the migrations of humans over several hundred thousand years. The p53 protein plays important roles in DNA strand break repair and, therefore, in the surveillance of aberrant DNA recombination, leading to the B-cell translocations that are causal in the pathogenesis of MM. We posit that polymorphisms in one region of the *TP53* gene (introns 2 and 3, and the proline-rich domain) specify a concentration of the p53 protein with a higher capacity to repress translocations in CA than AA patients. This, in turn, results in a higher risk of acquiring inactivating, somatic mutations in a different region of the *TP53* gene (DNA binding domain) in CA than in AA patients. Such a mechanism, by which the polymorphic status of a gene influencing its own “spontaneous” mutation frequency, may provide a genetic basis to address ethnicity-related differences in the incidence and phenotypes of many different forms of cancer.

## Facts

- There are phenotypic differences between the same tissue specific cancers of African and European ancestry.
- Polymorphisms that developed between African and European ancestry could regulate these differences.
- Some of these polymorphisms are located in introns and exons of the proline-rich domain of the *TP53* gene.
- These polymorphisms could regulate the frequencies of spontaneous mutations in the *TP53* gene found in a variety of cancers. Is this an example of a polymorphism in a gene regulating its own mutation rate?

## Open Questions

- What are the mechanisms of the polymorphisms regulating the rates of p53 mRNA synthesis, mRNA stability and p53 protein stability in different cell types of African and European ancestry?
- Do the concentration differences of the p53 protein in cells that develop cancers account for the different levels of spontaneous *TP53* mutations in the DNA binding domain of the protein?
- What epigenetic modifications of the p53 protein play a role in modifying p53 transcriptional activity resulting in differences in cancers of African and European ancestry?
- Do the selection pressures that regulate the development of polymorphisms that differ between the *TP53* genes in African or European descent reflect the role of p53 in the innate immune system? In the adaptive immune system?
- What percentages of the differences between cancer phenotypes in patients of African and European ancestry are contributed by these *TP53* polymorphisms?
- What mediates the differences between the phenotypes (the frequency of *TP53* mutations) in multiple myelomas and non-small cell lung cancers in different African and European ancestries?

## The development and statistics of multiple myelomas

Multiple myeloma (MM) is a cancer of the plasma cells in the body. These cells normally synthesize antibodies at a very high rate that are employed to protect us against infections. The precursors of plasma cells are B-cells (bone marrow derived cells) which engage an antigen, multiply, and differentiate into plasma cells to produce the antibodies. Malignant cancers of plasma cells are called MM. They are the second most common hematological malignancies in the USA, with 34,470 new cases per year and 12,100 deaths per year (in 2022, https://seer.cancer.gov/statfacts/html/mulmy.html). In the United States, there are approximately 160,000 cases of MM being treated by a variety of new and useful drugs. The median age at diagnosis is about 65–69 years and 5-year survival (2010–2018) is 58.7%. This cancer causes damage to bone, red blood cells, and the kidneys, with lots of protein (antibodies) in the blood and urine that are produced by the tumor cells. The rapid diagnosis of this cancer is a blood or urine test that detects abnormally high concentrations of paraproteins (monoclonal immunoglobulin fragments or intact immunoglobulins) resulting from the expansion of a single clone of plasma cells. The confirmation that very high levels of protein in the blood or urine detects a MM is an electrophoresis assay that demonstrates the very high levels of antibody. A PCR test sequencing the hypervariable region of the antibody gene producing these high levels demonstrates the clonality of the B-cell malignancy [1, 2].

There are at least two benign precursors of this cancer. Monoclonal gammopathy of undetermined significance (MGUS) consists of a benign growth and expansion of a clone of B-cells in the bone marrow and blood, producing a single antibody type at high levels. MGUS is present in about 1.7% of the CA population at 50 years and increases to over 8% at age 80 years. About 20% of MGUS progresses to Myeloma over time (the diagnostic tests are the same). It does so through a second precursor stage called smoldering multiple myeloma (SMM), which converts to MM with a frequency of 10% in the first year, 3% over the next 5 years, and 1–2% over the following 10 years [3–5].

What is particularly striking about MGUS, SMM, and MM is that 2/3rds of all cases in the USA occur in Americans of African descent (AA) and only 1/3rd occur in Americans of Caucasian descent (CA) when these groups are compared in equal numbers or population sizes. The AA population represents 13.4% of the U.S. population.

The annual rate of new MM cases (incidence) is at least twice as high among AA (males 17.0 and females 12.9 × 10^5^ person/years) as it is among non-Hispanic whites 8.1 and 5.0 × 10^5^ person/years in males and females, respectively (https://seer.cancer.gov/statfacts/html/mulmy.html) [6]. AA Individuals also commonly develop MM at younger ages, about 3–8 years earlier than CA patients [6]. As a consequence the age-adjusted prevalence rate of MGUS is three-fold higher in AA compared with CA. However, the estimated cumulative risk of MM developing from the benign precursors is similar for AA (17%) and CA (15%), suggesting that the excess risk of MM in AA results from an increase in the risk of MGUS rather than an increased risk of progression from MGUS to MM [3, 6]. These observations are consistent with the idea that the inherited input in the development of MM acts at the formation of MGUS, whereas spontaneous mutations might act at the transition from benign MGUS to malignant MM stage [3, 4, 6, 7].

In addition to these ethnic variations, several observations suggest that there are additional inherited components to at least some MM [7–9]. There is a 2.6-fold increase in MGUS in individuals with first degree relatives who have developed MM in the past. Current Genome Wide Association Studies have identified a large number of *loci* as significantly associated with MM risk, pointing out the involvement of four interconnected mechanisms: regulation of cell cycle and genomic instability, chromatin remodeling, *IRF4-MYC*-mediated apoptosis/autophagy, and B cell and plasma cell differentiation [7, 8]. In cases of familial aggregation, one particular antigenic target of paraproteins (so-called “paratargs”), namely the hyperphosphorylated form of paratarg-7 (pP-7) is frequently identified in affected members of high-risk MM families. pP-7 is an antigenic product of the STOML2 gene (8., Stomatin-like 2, 9p13), encoding a protein thought to regulate mitochondria biogenesis and activity. The frequency of pP-7 as an antigenic target appears to be particularly high in AA patients with MM. How such genetic variations could be contributing to the observed AA disparities in MM incidence is currently unknown [7, 8].

The global incidence of MM has increased by a striking 126% from 1960 to 2016, and deaths have increased by 94% during that time period. Some of this is likely due to enhanced longevity over the last fifty years. There are also enhanced methods of diagnosis. It is only in the last ten years or so that effective treatments for this disorder have appeared [2] and the lifespans and prolonged treatments of these patients have been increased. Access to enhanced diagnosis and new treatment is constrained by social disparities, which may exacerbate the striking ethnic difference in MM incidence. In a recent review of clinical trials for new drugs for MM (19 trials for 10,157 patients) 87% of the patients were CA, 7% were Asian American, and only 4% were AA [6, 9]. There is good evidence that this kind of bias leads to multiple disparities in obtaining good statistics, because of the problem of testing drug outcomes in small monolithic populations. The causes for this include a combination of issues: genetic differences, different disease biology, comorbidity conditions, access to care, access to novel treatments, and access to clinical trials. The result is that we do not actually know which drugs act differentially upon AA compared to CA [6, 9]. To address this we need to understand the mechanisms and genetic elements that differentiate African and Caucasian Americans.

## The molecular genetic basis for multiple myeloma

DNA sequencing studies from tumor and normal cells provide evidence for a genetic basis of MM ethnic disparities. For example, one study analyzed the sequence of MM in patients from CA, compared to AA, along with patients from Africa (Ghana) [6]. The frequencies of MGUS and MM were similar for AA and Ghanaians. In addition, the types of the molecular genetic alterations observed in Ghanaian and AA populations were closely related, and had some significant differences from CA, as determined by the spontaneous mutational profiles of three types of translocations (chromosome fusions) found to be associated with this cancer: t(11:14), t(14:16) and t(14:20), which are more common in Africans and in AA than in CA [7, 10].

Large-scale genetic analysis of MM cells showed that the most common genetic *locus* involved in MM patients is the IgH (immunoglobulin heavy chain) enhancer-promoter, which drives the transcription of several oncogenes through a set of translocations found in the benign (MGUS) and malignant (MM) tumors. The frequencies of IgH translocations initiating on human chromosome 14 are approximately 50% in MGUS, 60% in Intramedullary MM (where intramedullary denotes inside the bone marrow), 80% in Extramedullary MM (outside the bone marrow), and 90% in MM, demonstrating the progressive number of oncogenes recruited with increasing malignancy. These translocations are commonly fused with the *FGFR3*, *MMSET, CCND1, CCND3, MAF* or *MAFB* oncogenes located on other chromosomes. Malignant MM also commonly contains somatic mutations in *K- RAS*, *N-RAS*, *c-MYC* and *FAM46C* (12.6% in AA and 8.3% in CA), *BRAF-V600E* (0.8% in AA and 4% in CA; of note, this may result in treatment decision differences) and deletions in chromosome 13q [10–12].

**Fig. 1:**
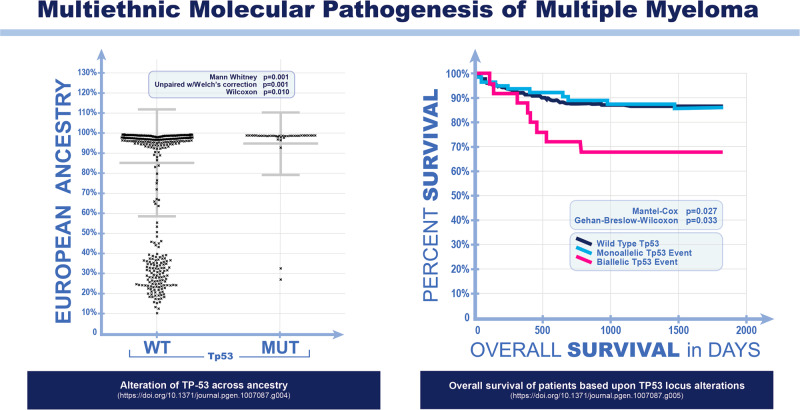
Multiethnic molecular pathogenesis of multiple myeloma: TP53 mutations identify a subset of MM with very poor outcomes in terms of overall survival. Exonic sequencing was carried out with approximately 150 multiple myelomas (80% purified from bone marrow) of African and European ancestry and germ line sequences from normal cells of patients (see ref. [11] for the original results reported for this experiment and modified for this figure). This permitted the assignment of spontaneous *Tp53* mutations which are reproduced here from ref. [11]. Clearly there is a preference for *Tp53* mutation in patients of European ancestry and those patients have a phenotype (poorer overall survival) if there is a bialleic *Tp53* mutation.

Differential spontaneous somatic mutation profiling in CA versus AA MM cases has revealed that the most striking and statistically significant difference between the two groups lies in the prevalence of somatic mutation in the *TP53* tumor suppressor gene [11]. Specifically, *TP53* is somatically mutated at a frequency of 6–8% in CA MM cases compared to 0–1.6% in AA MM cases. In addition, *TP53* mutations identify a subset of MM with very poor outcomes in terms of overall survival [11] (Fig. 1). Tumor suppression is a phenotype that leads to a selection for *TP53* mutations in cancer cells, and this appears to occur in MM predominantly in Americans from European descent. Thus, the association between *TP53* mutations and poorer outcome is consistent with the role of wild-type p53 protein acting as a stronger tumor suppressor in MM from CA patients compared to AA patients. Notably, there are fewer cases of MM observed in CA compared to AA individuals, adjusted to the population sizes. When CA MM patients have *TP53* homozygous mutations, their overall outcomes are three times worse than for CA patients without *TP53* mutations [11]. Importantly, *TP53* is highly polymorphic, with several functionally-relevant polymorphisms showing significant variations in relation to ethnicity (with and without spontaneous *TP53* mutations) [13]. These combined observations suggest that inherited variations in the *TP53* gene (polymorphisms) between AA and CA Americans contribute to the differences discussed above in the incidence of MGUS and SMM, and that spontaneous *TP53* mutations may differentially occur on distinct *TP53* haplotypes, conferring new phenotypes to MM among these populations.

## The functions of the p53 protein

The p53 protein is a transcription factor regulating hundreds of genes, many of which contribute to multiple aspects of tumor suppression [13]. The protein is activated to transcribe genes by a variety of stresses resulting in epigenetic modifications (inhibition) of a p53 ubiquitin ligase, MDM-2, which initiates the degradation of the p53 protein. DNA damage is a common stress that activates p53 for transcription, which then decides either to enter cell cycle arrest and repair the damaged DNA or to eliminate the damaged cell via five different cell death mechanisms [13]. Replication of damaged DNA often causes mutations, and wild type p53 prevents or lowers the frequency of mistakes resulting from inappropriate repair of DNA breaks. Inactivating *TP53* mutations, mostly in the DNA binding domain (amino acids 102–292) of the p53 protein, impair p53 transcriptional activity and contribute to the genetic instability that fuels cancer growth. About 50% of all human cancers contain mutations in the DNA binding domain of the p53 protein. Other mechanisms (such as MDM-2 amplification, PPMID mutation, inactivation of cell death pathways) can result in the indirect loss of p53 activity and tumor suppression, resulting in at least to 80% of the human cancers having dysfunctional p53 pathways [13].

The specific functions of p53 in DNA strand break sensing and repair bring up a potential problem for those cell types, B-cells, T-cells, and germ line cells, that undergo high levels of recombination as they differentiate normally and produce products central to other life functions or reproduction. The breaks in the DNA, as part of the recombination process, should cause p53 activation and either kill these cells or assist in their repair and survival. So, it is likely that the functions of the p53 protein need to be tightly controlled during recombination, and at least partially inactivated or tamed to prevent counterproductive cell destruction [14, 15]. Several questions arise: How is this accomplished? If p53 inactivation occurs, why don’t these tissues undergo a much larger number of mutations and cancers? Does this process of controlling inactivation of p53 differ between AA and CA? Do the *TP53* gene and protein constitutively differ between AA and CA Americans? (the answer is yes). Do genetic differences affect the development of MGUS between CA and AA and do they map to the *TP53* gene? [13]. Are these differences related to the mechanisms of *TP53* inactivation (mutations are found preferentially in CA) during B-cell development? Piecing together the evidence from epidemiology, clinical phenotypes, genetics and studies on p53 functions suggests that, in CA, wild-type p53 may operate as a stronger suppressor of inappropriate DNA strand break recombination and repair than in AA. This, in turn, would explain why [1] translocations causing MGUS are more frequent among AA compared to CA, and [2] the selection pressure for inactivating *TP53* by somatic mutation is higher in CA than in AA, causing the large number of differences observed in the frequencies of these mutations between the two populations.

Answers to many of these questions may well be in the literature, but key experiments remain to be done to provide definite proof of the mechanisms which underlie the basis of the different cancer phenotypes in AA and CA patients. Solving these questions has fundamental clinical implications, given that the drugs that have been developed to treat MM may not function equally in AA and CA patients. So far, this question has not been analyzed properly in those two groups [6].

The Origin and Nature of Genetic Differences in the *TP53* Gene of Americans of African and European Descent

The p53 protein is assembled from 11 exons encoding 393 amino acids [13]. The gene is highly polymorphic, with hundreds of single nucleotide polymorphisms (SNP) in strong linkage disequilibrium, specifying well-defined haplotype blocks that have evolved as genetic units [16]. Three of those SNPs specify differences in the amino acid sequence of the p53 protein derived from African or European descent: [17–21] p.P47S (c.139 C > T, proline to serine at codon 47, SNP link 1800371), p.R72P (c.215 C > G, arginine to proline at codon 72, SNP link1042522) and p.Y107H (c.319 T > C, tyrosine to histidine at codon 107, SNP link368771578) (see Table 1 where these SNPs have African or European origins). Two of them, codons 47 and 107, are rare variations (2% to <1%). The functional significance of a fourth variant, p.V311is unknown. Having serine at amino acid 47 (p.S47) causes impaired phosphorylation on serine 46, which is critical for the ability of p53 to induce apoptosis; consequently, p.S47 is associated with impaired apoptosis [19, 20] and increased risk for early stage breast cancer in AA women [19]. P.S47 is also associated with impaired ability to undergo ferroptosis [20], impaired ability to recognize and bind to sites of DNA damage in cells and increased activation of mTOR, a master regulator of metabolism [20]. On the other hand, P.Y107H is present in 0.1% of African-descent individuals. This form of p53 shows impaired ability to transactivate the epigenetic modifier PADI4, which controls immune recognition of tumors [21] (Table 1). Based upon these phenotypes these rare polymorphisms could contribute to some differences in AA and CA cancer phenotypes.

**Table 1:** Is There Evidence for Differences in the *Tp53* gene in multiple myelomas of Americans of African versus European descent?.

Codon	African Descent	European Descent	Phenotypes
31			?
47	serine	proline	Impaired apoptosis, ferroptosis and increased m-TOR activity
72	proline	arginine	LFS- earlier age of onset of Cancers; Breast cancer differences in overall survival, MDM-2-p53 binding site
107	histidine	tyrosine	Cancer survival, immune responsiveness P53 responsive gene-*PADI-4* regulates the immune response

Intronic polymorphisms		
Pin2	G/C polymorphism in intron 2	RNA levels regulated
Pin 3	16 bp duplication/16 bp	Splicing variations and a G-quadraplex, RNA levels regulated
(refs: exonic, 18–22; intronic, 32, 33)		

Three exon coding region polymorphisms have been identified in the *Tp53* gene from individuals of African descent and European descent. These amino acid changes are as follows (the p53 protein is composed of 393 amino acids).

**Fig. 2:**
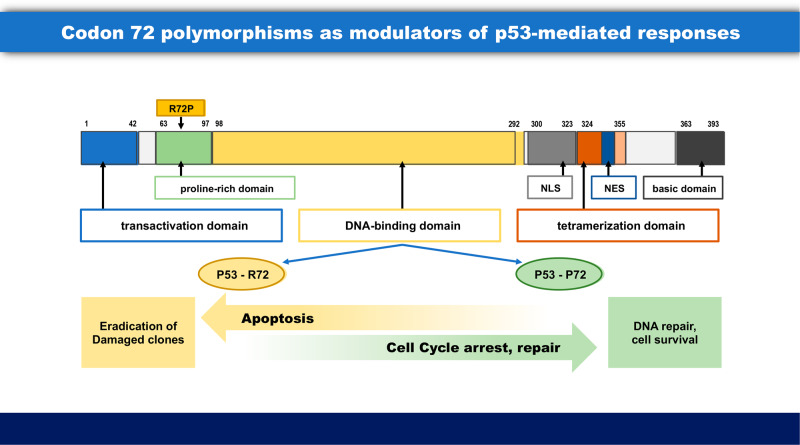
Codon 72 polymorphisms as modulators of p53-mediated responses: In response to DNA damage, the R72 variant is more effective at inducing apoptosis, whereas P72 is more effective at mediating cell cycle arrest and repair. As a result, each variant may respond to different selection pressures, depending upon cell and tissue context. In the context of MGUS- > MM progression, the R72 variant is more effective than P72 at eliminating single cells with excess/abnormal recombination, thus preventing the occurrence of initiating oncogenic translocations that cause MGUS. This effect is fully eliminated by *TP53* mutation. In the context of epithelial cancers (breast; colon), the P72 variant is more effective than R72 at repressing cell proliferation across the tissue therefore impairing physiologic competence in the face of persistent mutagenic exposure. This effect is fully eliminated by *TP53* mutation that enables surviving initiated tumor cells to progress towards aggressive cancer.

In contrast to these rare SNPs (p.P47S and p.Y107H), p.R72P is a very common variation. The G allele (encoding R) is found in 60–80% of Caucasians, whereas the C allele (encoding P) is present in 65–75% of Africans. Worldwide, the distribution of p.R72P appears to be under natural selection as a function of latitude (from the equator north or south). Specifically, from the equator to Scandinavia, codon 72 increases in frequency from P to R in the populations migrated out of Africa to northern Europe [22, 23]. This variation occurs within a structural proline-rich, SH-3 binding domain of the p53 protein, so that substitution of R for P in codon 72 of this domain modulates the interactions between p53 and cellular proteins binding to this domain [22]. The p.P72 variant binds with decreased affinity to MDM2 [24, 25], resulting in increased levels of p53 in the cell, and an impaired ability to take part in transcription-independent mitochondrial pathway to cell death [26–28]. In vitro studies with human cancer cells and rodent fibroblasts have shown that following DNA damage, the P72 variant preferentially promotes cell cycle arrest, senescence, and DNA repair, whereas the R72 variant more effectively induces apoptosis [26–28] (Fig. 2). Epidemiological studies have shown modest, but in some cases significant, associations of p.R72P variants with the expected properties of the codon 72 genotypes and phenotypes in cancers. In individuals of African and European descent with non-small cell lung cancers, there were significantly larger levels of the p53 protein in tumor cells of codon 72 P/P homozygotes than P/R or R/R carriers [29]. This was expected, because the binding constant of the ubiquitin ligase MDM-2 to p53, P72 p53 is weaker than to p53 R72 [25]. Under these conditions, higher levels of wild type p53 in a cell, that are P/P at codon 72, would exert greater selection pressure to reduce tumor suppression as cancers form, resulting in an increased number of spontaneous mutations (in codons 102–292) causing the loss of transcription by p53. In a publication examining the number of *TP53* mutations in a population of NSCLC patients, there were a greater number of *TP53* mutations in the DNA binding region of the p53 protein in codon 72 P/P homozygotes (65%), than heterozygotes, R/P (57%) and R/R homozygotes (40%), *p* = 0.01 [30]. This is a very unusual configuration, where a polymorphism in a gene enhances the frequency of spontaneous mutations at other sites in the same gene.

Overall, there is good evidence that the codon R/P variation has evolved as a mechanism that modulates the adaptation of *TP53* functions to different ecological contexts. The environmental variables that are associated with changes in latitude are UV light, altitude, temperature and pigmentation (and possibly others not yet uncovered). Mutations that occur in the proline-rich region of p53 are enriched in skin cancers [22]. The changes in amino acids at codons 47, 72, and 107 do carry phenotypic associations (apoptosis and ferroptosis, binding affinity of the p53 protein to MDM2 ubiquitin ligase regulating p53 protein levels, SH3 protein binding affinities, metabolic differences) that have an impact on the frequencies of cancers [19, 22]. These polymorphic alleles are not oncogenes or tumor suppressor gene activities by themselves, rather they are modifiers of cancer incidence, severity, and biological properties.

**Fig. 3:**
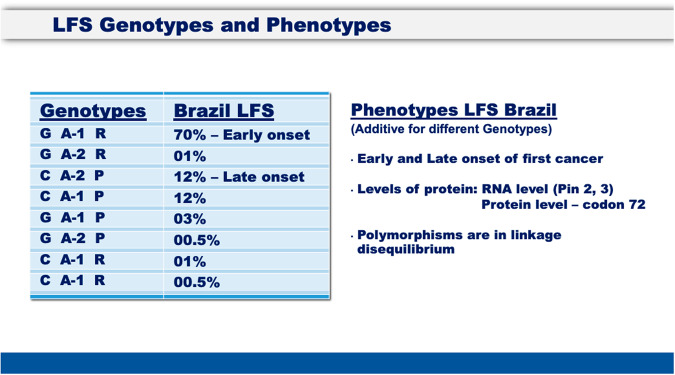
The eight genotypes and phenotypes of Pin 2, Pin 3, and Codon 72: There are eight genotypes presented in the figure, with their frequencies and their phenotypes, which are early or late onset of first cancers. These genotypes are not independent, but dependent upon all three polymorphisms. These three polymorphisms are additive for the different genotypes, giving rise to the early onset phenotype, which is up to 19 years earlier first cancers in LFS.

The region of the protein that specifies the proline-rich region, including residue 72, is encoded by a portion of the *TP53* gene that constitutes a short haplotype block encompassing exons 2, 3, and 4, with their intervening introns. This portion of the gene is made up of a succession of short introns and exons over less than 500 bp [16]. In addition to p.R72P, it contains two frequent intronic polymorphisms that are in strong linkage disequilibrium with p.R72P (G/C intron 2, SNP link 1642785 and 16 bp duplication in intron 3, SNP link 17878362) [31, 32]. Whereas p.R72P modulates p53 protein functions, there is evidence that both intron 2 and 3 polymorphisms influence p53 mRNA synthesis, splicing and stability via a G-quartet at that site (Table 1) [31, 32]. These three genetic *loci*, at codon 72 and intron 2 and 3, contain variations within this short region that appear to cooperate to specify a *TP53* haplotype with different expression dynamics, biological activities, and frequencies among individuals of Caucasian vs. African ancestry [31, 32]. Supporting this hypothesis, Brazilian carriers of germline *TP53* mutations (at risk for Li-Fraumeni Syndrome, a familial predisposition to multiple early cancers) have an accelerated or a delayed age of onset of first cancers depending upon the *TP53* alleles p.R72P, Pin2 (G/C) and Pin3 (A-1 or A-2) present in each patient (see Fig. 3) which together can affect the age of a first cancer [31, 32]. The available evidence suggests it is the sum of the three interacting genotypes (G/C, A-1/A-2, R/P) that give rise to the sum of the biological properties (p53 levels) of these polymorphisms that may well function as modifiers that distinguish the phenotypic differences between AA and CA cancers. The fact that these polymorphisms are in linkage disequilibrium supports the idea that they function together (Fig. 3). This set of observations [33–35] helps to explain why the codon 72 polymorphism, by itself, has provided some contradictory results in the literature. It is not p.P72R by itself that provides the phenotype, but the integrated haplotype structure (the addition of these three polymorphisms) that contribute several common polymorphisms (at the RNA stability and protein degradation levels) that provide the best (to date) predictive information. It is likely that additional inherited and environmental variables will be uncovered in the future that will be shown to have an impact upon the differences between AA and AC cancers.

## Summary

Integrating epidemiology, pathology, and genetics of MM with p53 functions highlights a series of interesting correlations: 1. MM occurs in the United States much more frequently in individuals of African descent than of European descent, when adjusted for relative population size. 2. Many of the causal mutations in MM originate from translocations (recombination) in B-cells with DNA breaks joining the IgH enhancer promotor to an oncogene. 3. These kinds of breaks in the DNA, which are required for B-cell function, are usually detected by the p53 protein and are either repaired or the cell is killed, preventing the fixation of oncogenic translocations. 4. Inactivating mutations in the *TP53* gene occur in excess (at least 4–6-fold higher) in CA versus AA patients. 5. There are significant differences in the amino acid sequences of the p53 protein between CA and AA individuals. In particular, the two populations differ in the structure of a haplotype block encompassing codon 72 (p.R72P) known to modulate p53 functions in response to DNA damage.

The questions raised by these correlations are: does this imply that the functions of the p53 protein in the B-cell lineage of AA individuals differ from CA individuals? Do these differences explain the excess incidence of MM in patients of African descent?

### Possibilities

The observations reviewed here demonstrate that MM occurring in patients of AA versus CA descent differ and that a significant candidate for mediating these differences is the *TP53* gene. This gene has evolved distinctive polymorphisms in individuals from Africa and from Northern Europe. These polymorphisms are candidates as genetic and epigenetic susceptibility factors for MM development. They may well entail phenotypes that influence the age of onset of MGUS and MM occurrence and its clinical outcomes (overall survival). These polymorphisms occur at frequencies that are reasonably high (R72P exists in ~65% of African Americans, and P47S occurs in 1–2% of African Americans). Interestingly, in Caucasian MM patients [33], the PP and PR genotypes at codon 72 (in combination) are more frequent among cases than in controls (65% vs. 42%, OR = 2.32, *p* = 0.04) of MM [33]. This suggests that, aside from other ethnicity-related genetic variations between AA and CA individuals, the P72 allele of *TP53* is likely to be a contributor to the age of onset and the risk of developing MM. What makes these polymorphisms special is that they also operate as susceptibility factors for the acquisition of spontaneous somatic, inactivating mutation in the *TP53* gene, which appears to contribute to overall survival. A recent meta-analysis has shown that cancer-risk associated germline variants may interact with somatic *TP53* mutational status to modify cancer risk, progression, and response to therapy in a range of different cancer types [34, 35]. Here, we suggest that such interactions may involve cooperative mechanisms by which these polymorphisms control the selection of somatic inactivating *TP53* mutations in a haplotype-specific manner. Higher levels of the p53 protein in a cell place a greater selection pressure upon the *TP53* gene to be a mutant *TP53* gene in a cancer.

One of the more important issues raised by this perspective is that drug trials commonly do not stratify the patients tested with a particular drug for genetic variables that could affect the efficacy of that drug for one group but not the other group. That may be the case for MM. However, one needs to know what genetic or environmental variables to compare a drug treatment with, before a clear result can be understood (ref. [33] is a good example of this). In support of this premise, in animal models and in pre-clinical studies, the p.P47S and p.Y107H variants decrease the efficacy of certain genotoxic chemotherapeutics [21, 34], and the p.P47S variant decreases the efficacy of immune checkpoint inhibitors [35].

The concept of cooperative interaction between *TP53* haplotypes and somatic mutation may not be restricted to MM. Several other cancer tissue types that do not normally undergo recombination during differentiation, have been shown also to differ between AA and CA patients. Prostate, colon, basal cell breast cancers, lung, and gastric cancers have all been shown to have significantly different characteristics in these two populations of patients [36, 37]. In some cases, the *TP53* gene, and in particular the codon 72, P/R polymorphism, as well as other SNPs in linkage disequilibrium, have been implicated as important variables in these differences [28–33]. To test these ideas, one will have to make the measurements of p53 activity and levels in cells with different genotypes and different cell types. This explanation of how combined SNPs and haplotypes can regulate MDM-2 binding to p53, which in turn can regulate p53 levels and activities, which in turn can regulate the selection pressure for *TP53* spontaneous mutations in a cell (mutation incidence), and even the age of onset of a tumor initiated by a *TP53* mutation, is a reasonable way to understand the phenotypes discussed in this manuscript. There is, however, enough heterogeneity in the results of such studies in the literature to suspect that additional polymorphisms or environmental variables contribute to the differences that are reproducibly observed between these cancers in AA versus CA patients.

Finally, there is likely a fundamental difference between the regulation of B-cell, T-cell, and germ line development and tumorigenesis compared to most other tissues, such as prostate, colon, breast, and lung. The former all undergo an active stage of recombination and double strand breaks as part of producing their fully differentiated products, whereas the latter do not. One of the major functions of the p53 protein is to respond to the detection of double strand breaks (by ATM and CHEK-1,2) and decide to either kill the cell or repair the break. If p53 were to execute these tasks too efficiently there would perhaps be no surviving B-cells, T-cells, nor germ line. Thus, the p53 protein must be regulated differently (attenuated or switched off) in cells that undergo recombination. Interestingly, the incidence of cancers of these three cell types also differs in different ethnic groups. In MM, AA have 2 times higher incidences of this cancer than CA. The incidence of ALL and CLL each differ when the age-dependent incidence of CA and AA are compared [38]. In testicular teratocarcinomas, the incidences of cancers are: CA, 11.5/100,000 individuals; Asians: 1.5 cases/100,000; AA: 0.3 cases/100,000 [39]. In these three cancer tissue types, the ethnic groups with the highest or lowest incidences of cancer differ from each other in different tissues, so the mechanisms that mediate these incidences of cancer might well differ because of different tissue characteristics. Indeed, that appears to be the case. In testicular teratocarcinomas of individuals of Caucasian ancestry, the embryonal carcinoma stem cells (EC cells) lack *TP53* mutations, (the opposite of MM). This is because the EC cells produce a p53 protein that is not a transcriptionally active [40] so there is no selection pressure to produce mutations in the tumor. The inactivation of the p53 protein in EC cells is mediated by epigenetic modifications, by methylation of three lysine residues ((Smyd2:K379me; GLP/G9a:K373me; Set8/Prset7:K382me) in the p53 protein) [41]. In this case epigenetic modifications of proteins regulate the activities of the p53 protein, which is a common feature of phenotypic differences. This is consistent with the idea that the same genotypes may be treated differently (with epigenetic modifications) in different cell types giving rise to different phenotypes in those different cell types. There are some examples of this in this review. In addition, there is good evidence that MGUS and subsequent MM in AA results from a combination of genetic and environmental susceptibility factors [42]. Determining just what those factors are is an important step in treating this disorder.

Thus, there appear to be several different mechanisms that may contribute to differences between AA and CA patients in tumor formation and outcomes, depending upon the environment, the tissue type, and the biological and epigenetic, protein modifications that play a role. It appears, however, that some of these processes can be traced back to the evolution of the *TP53* gene and protein, which respond to environmental changes and the migrations of populations. The ideas presented here can be tested, and we hope will permit new avenues of investigation producing the most appropriate treatments for cancers of different tissues in different individuals.

### Supplementary information

checklist
